# Incidence and Mitigation of Corneal Pseudomicrocysts Induced by Antibody–Drug Conjugates (ADCs)

**DOI:** 10.1007/s40135-024-00322-5

**Published:** 2024-03-24

**Authors:** Ethan S. Lindgren, Rongshan Yan, Onur Cil, Alan S. Verkman, Matilda F. Chan, Gerami D. Seitzman, Asim V. Farooq, Laura A. Huppert, Hope S. Rugo, Paula R. Pohlmann, Janice Lu, Laura J. Esserman, Neel D. Pasricha

**Affiliations:** 1 Department of Ophthalmology, University of California San Francisco, San Francisco, CA, USA; 2 Department of Pediatrics, University of California San Francisco, San Francisco, CA, USA; 3 Departments of Medicine and Physiology, University of California San Francisco, San Francisco, CA, USA; 4 Francis I. Proctor Foundation, University of California San Francisco, San Francisco, CA, USA; 5 Department of Ophthalmology and Visual Science, University of Chicago Medical Center, Chicago, IL, USA; 6 Helen Diller Family Comprehensive Cancer Center, University of California San Francisco, San Francisco, CA, USA; 7 Department of Breast Medical Oncology, University of Texas MD Anderson Cancer Center, Houston, TX, USA; 8 Department of Medical Oncology, Keck School of Medicine, University of Southern California, Los Angeles, CA, USA; 9 Department of Surgery, University of California San Francisco, San Francisco, CA, USA

**Keywords:** Antibody–drug conjugates, Corneal pseudomicrocysts, Microcyst-like epithelial changes, Ocular surface adverse events, Ocular surface epithelium, Cornea, Conjunctiva

## Abstract

**Purpose of Review:**

This study is to highlight the incidence of corneal pseudomicrocysts in FDA-approved antibody–drug conjugates (ADCs), and success of preventive therapies for pseudomicrocysts and related ocular surface adverse events (AEs).

**Recent Findings:**

ADCs are an emerging class of selective cancer therapies that consist of a potent cytotoxin connected to a monoclonal antibody (mAb) that targets antigens expressed on malignant cells. Currently, there are 11 FDA-approved ADCs with over 164 in clinical trials. Various AEs have been attributed to ADCs, including ocular surface AEs (keratitis/keratopathy, dry eye, conjunctivitis, blurred vision, corneal pseudomicrocysts). While the severity and prevalence of ADC-induced ocular surface AEs are well reported, the reporting of corneal pseudomicrocysts is limited, complicating the development of therapies to prevent or treat ADC-related ocular surface toxicity.

**Summary:**

Three of 11 FDA-approved ADCs have been implicated with corneal pseudomicrocysts, with incidence ranging from 41 to 100% of patients. Of the six ADCs that reported ocular surface AEs, only three had ocular substudies to investigate the benefit of preventive therapies including topical steroids, vasoconstrictors, and preservative-free lubricants. Current preventive therapies demonstrate limited efficacy at mitigating pseudomicrocysts and other ocular surface AEs.

## Introduction

Antibody–drug conjugates (ADCs) comprise a growing category of targeted cancer therapy [1••, 2••]. The first ADC was approved in 2000 for acute myeloid leukemia (gemtuzumab ozogamicin, Mylotarg) [3]. Today, there are 11 FDA-approved ADCs on the market designed to treat hematological malignancies and solid tumors, with an additional 164 in the clinical trial pipeline [4–7]. In concept, ADCs offer increased selectivity towards cancer cells while minimizing the systemic and off-target toxicities accompanied by traditional chemotherapies [8, 9]. Despite the designed selectivity of ADCs, adverse events (AEs) are still common in various tissues, including the eye [9]. The most prevalent forms of ADC-related ocular surface AEs are keratitis/keratopathy, dry eye, conjunctivitis, blurred vision, and corneal pseudomicrocysts, previously known as microcyst-like epithelial changes (MECs) [10]. This review focuses on the prevalence and medical management of corneal pseudomicrocysts in addition to other ocular surface AEs.

**Fig. 1:**
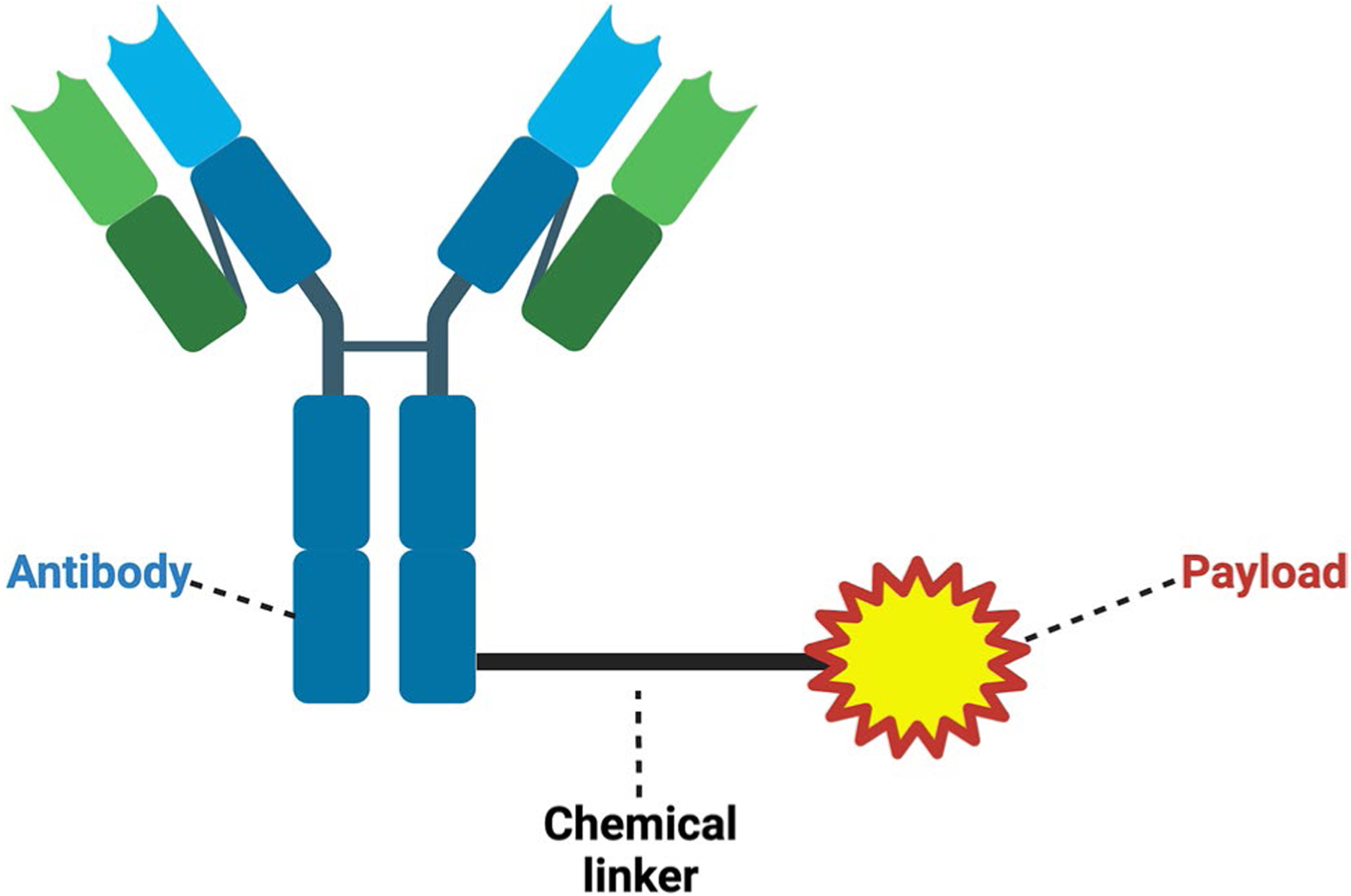
Structure of ADCs. There are three well-defined components: antibody, chemical linker, and cytotoxic payload. Of the current FDA-approved ADCs, all have IgG1 or IgG4 mAbs. Payloads, which either damage DNA or disrupt microtubule formation during mitosis, are linked to the mAb via a chemical linker, which are defined as cleavable or non-cleavable. Created with BioRender.com

An ADC is composed of a monoclonal antibody (mAb) that is fused to a highly potent cytotoxic payload by a chemical linker (Fig. 1). mAbs bind to an antigen expressed on tumor cells enabling the efficient delivery of the cytotoxic payload to the tumor [11]. Each component of an ADC has the potential to play a role in toxicity [11–13].

ADC treatment is typically administered every 1 to 4 weeks via 30-min intravenous infusion [14•, 15]. The success of ADCs can be hindered by resistance to ADCs or severe systemic AEs across the body that necessitates discontinuation of therapy [1••]. Resistance to ADCs may be related to the rate of ADC internalization, changes to antigen expression on the target cell, or resistance to the payload [16].

Ocular surface AEs are clinically relevant due to their ability to interrupt ADC treatment. One study evaluating the safety profile of belantamab mafodotin (Blenrep) found that 72% of patients developed corneal pseudomicrocysts. These pseudomicrocysts caused dose delays in 47% of patients and dose reductions in 25%. Corneal pseudomicrocysts necessitated that 3% of patients discontinue ADC infusions [17••]. These dose modifications highlight the importance of corneal pseudomicrocyst mitigation.

### Corneal Pseudomicrocysts

**Fig. 2:**
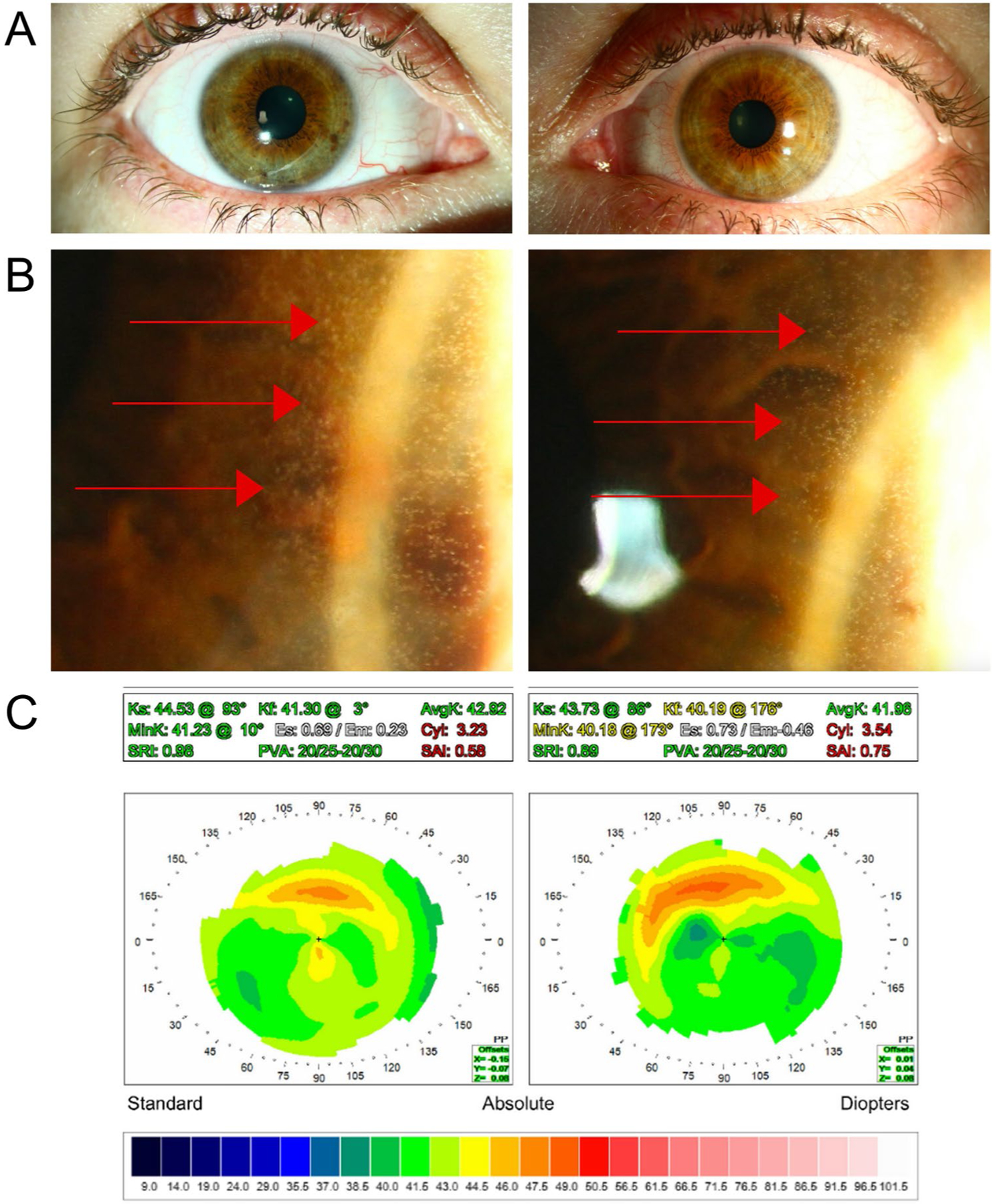
Manifestation of corneal pseudomicrocysts. **A** Examination of the ocular surface with diffuse illumination appears normal. **B** High magnification sclerotic scatter show numerous peripheral corneal pseudomicrocysts. **C** Corneal topography demonstrates peripheral ring-like steepening corresponding to the area of corneal pseudomicrocysts

Corneal pseudomicrocysts are microcyst-like structures located in the corneal epithelium’s basal layer [10]. Corneal histology suggests that these represent intracytoplastic inclusions within pre-apoptotic or apoptotic epithelial cells [18]. Corneal pseudomicrocysts initially emerge bilaterally around the limbus in a ring-like pattern and migrate toward the central cornea with subsequent drug infusions (Fig. 2) [10, 17••, 19, 20•]. Anterior segment optical coherence tomography (AS-OCT) and in vivo confocal microscopy (IVCM) can identify these structures as hyperreflective tiny circles. Notably, true cystic structures would be hyporeflective [20•, 21]. Patients with corneal pseudomicrocysts commonly report symptoms such as blurred vision, dry eye, irritation, tearing, and photophobia. However, some patients with corneal pseudomicrocysts can be asymptomatic [17••, 20•]. Peripherally located corneal pseudomicrocysts cause relative central corneal flattening and induce a hyperopic shift, while centrally located cysts cause relative peripheral flattening, leading to a myopic shift [22•].

Patients receiving ADC treatment may develop corneal pseudomicrocysts as early as 3 weeks after the start of infusions [22•, 23]. These corneal changes are reversible upon the discontinuation of treatment within a matter of weeks to months (2–32 weeks) [17••, 21, 22•, 24–26]. In our experience, some patients take up to 9 months for complete resolution of corneal pseudomicrocysts. However, resumption of ADC treatment can cause a rapid reappearance of corneal pseudomicrocysts that may take longer to resolve after ADC treatment stops [19]. The success of topical steroids, vasoconstrictors, and preservative-free artificial tears (PF-ATs) as prophylaxis or treatment for corneal pseudomicrocysts varies [17••, 27•, 28]. Dose reductions, delays, or discontinuations are currently the only known efficacious strategy for mitigation [2••, 10, 27•, 29, 30].

### Literature Review

Corneal pseudomicrocysts is the accepted current terminology for the cyst-like corneal changes induced by ADCs. However, the nomenclature for corneal pseudomicrocysts is inconsistent in the literature. This review specifically evaluates corneal pseudomicrocysts reported in the literature and summarizes preventive therapies. Publications were searched for on two databases: PubMed and Drugs@ FDA. Search terms included individual FDA-approved ADC names (including belantamab mafodotin) and various terms used interchangeably to characterize corneal pseudomicrocysts. These terms include the following: pseudomicrocysts, microcyst-like epithelial changes (MECs), microcyst epithelial keratopathies (MEKs), cornea cysts, corneal epithelial deposits, corneal epithelial microcysts, keratopathy, microcyst corneal lesions, and intra-epithelial opacities.

## Results

Table 1 summarizes the incidence and prevalence of the ocular surface AEs associated with ADC treatment. There are 12 ADCs listed, 11 of which are FDA-approved and used in clinical practice as of January 2024. Additional ADCs have received FDA-approval but have been removed from distribution for safety or economic reasons. Belantamab mafodotin (Blenrep) was previously FDA-approved but then withdrawn from the US market after failing to meet certain FDA requirements during phase III trials. Of the various ocular surface AEs, a special focus is given to corneal pseudomicrocysts. Three (25%) of the ADCs listed in Table 1 induced corneal pseudomicrocysts, and six (50%) were reported to induce other ocular surface AEs, of which dry eye was the most commonly reported (6, 100%). Keratopathy/keratitis was the second most common ocular surface AE (3, 50%) followed by conjunctivitis (2, 33%). Of the six ADCs with ocular surface AEs, only three (belantamab mafodotin, mirvetuximab soravtansine, tisotumab vedotin) had ocular substudies that evaluated the efficacy of preventive therapies for ocular surface AEs. Of these substudies, only one demonstrated that topical steroids could mitigate ADC-induced corneal pseudomicrocysts. Another found that a regimen of vasoconstrictors, topical steroids, and PF-ATs reduced the rate of conjunctivitis and ocular AEs.

### Mechanism of ADC-Induced Ocular Toxicity

Characteristics unique to both the ocular surface (cornea and conjunctiva) and ADCs are suspected to play a role in toxicity of ADCs [31, 32]. The ocular surface is likely susceptible to ADC-related toxicity due to a rapidly regenerating population of limbal stem cells, diversity of cell surface receptors, and rich blood supply [10, 11]. ADCs’ structure may contribute to toxicity due to linker instability or expression of the target antigen in ocular tissues [11]. Notably, of the three ADCs that induce corneal pseudomicrocysts, one has a target antigen that is expressed in both the cornea and conjunctiva (HER2), one has a target antigen expressed solely in the conjunctiva (FRα), and one has a target antigen that is not expressed on the ocular surface (BCMA) [33].

**Fig. 3:**
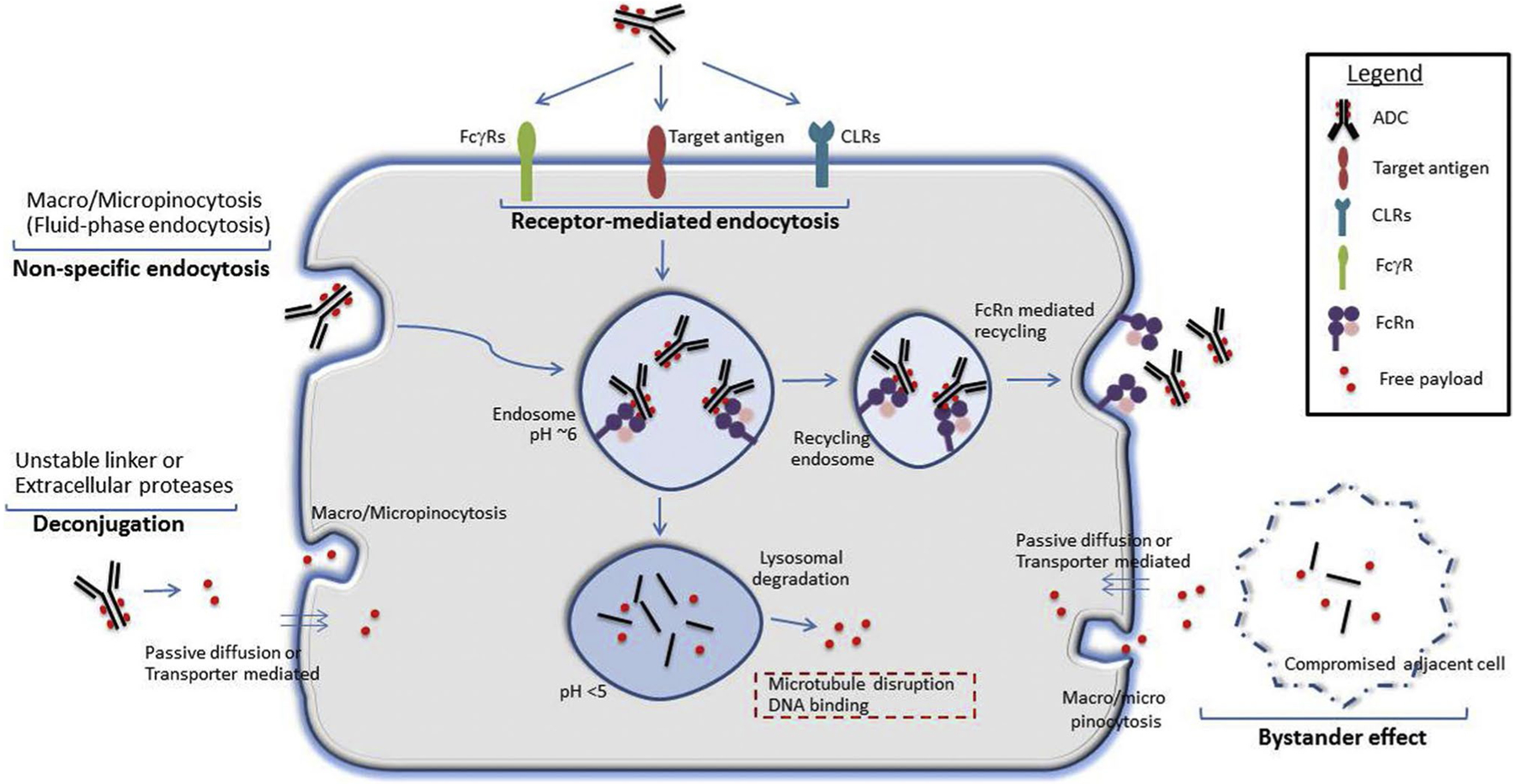
Potential mechanisms for ADC-induced ocular surface toxicity (reprinted with permission from Elsevier, originally published in Mahalingaiah et al. Potential mechanisms of target-independent uptake and toxicity of antibody–drug conjugates. Pharmacol Ther. 2019)

Off-target toxicity of ADCs on the ocular surface can be attributed to various mechanisms (Fig. 3). Macropinocytosis, a form of non-specific endocytosis known as “cell drinking,” facilitates the internalization of ADCs or the deconjugated payload [10, 31]. Fc and C-type lectin receptors enable endocytosis in a receptor specific manner [10, 31]. Internalization of an ADC in normal tissue can lead to the premature release of the cytotoxin due to linker instability or linker degradation by normal metabolism in the cell [23]. Additionally, the payload may passively diffuse into a cell due to non-charged, membrane permeable residues on the payload [34]. Another mechanism is the “bystander effect” where after an ADC is internalized and degraded by a cell, a fraction of the payload can release from the dead cell into the extracellular space and kill neighboring cells, regardless of their target antigen expression (35).

Drug-antibody ratio (DAR), or drug loading, is another significant player in ADC toxicity. Each antibody is attached to a certain number of payloads; FDA-approved ADCs range between DAR 2 and 8 [1••]. Mouse studies have demonstrated that a higher DAR has a lower therapeutic index [36]. In monkey studies, a higher DAR was associated with an earlier onset and a higher incidence of corneal toxicity [37•].

### Novel Preventive Therapies

Several preventive therapies have been investigated to mitigate ADC-induced corneal toxicity to varying success.

Topical steroid eye drops have been used with various ADCs because they are hypothesized to slow down limbal stem cell regeneration and, in theory, make the cornea less susceptible to toxicity [28]. Outcomes from steroid eye drops have ranged from no benefit to complete clearance of corneal pseudomicrocysts [28, 30, 38]. Variation in response to steroids can likely be attributed to biological variability in the patient population and differing mechanisms of toxicity [2••]. In our experience, topical steroid eye drops do not address the underlying corneal pseudomicrocysts but may alleviate some eye pain symptoms. Vasoconstricting eye drops and cold compresses were used to mitigate ocular AEs during infusions of Tisotumab vedotin (Tivdak) by reducing drug uptake in the cornea. Although vasoconstrictors reduced the rate of conjunctivitis, their effect on corneal pseudomicrocysts has not been reported [39]. An ocular substudy on the ADC depatuxizumab mafodotin (ABT-414), an EGFR mAb conjugated to the tubulin inhibitor monomethyl auristatin F (MMAF) via a stable maleimidocaproyl link, found no difference in the prevalence of ocular surface AEs in eyes treated with topical steroids with and without vasoconstrictors. This study also evaluated the use of a bandage contact lens for ocular surface AEs and observed no protective benefit, with nearly half the patients progressing to develop grade 3 ocular surface AEs [40]. PF-ATs can provide relief for certain ocular surface AEs, such as dry eye, but have no preventive effect for the pseudomicrocysts [2••]. Therapies like antihistamines are used to alleviate ocular AEs such as conjunctivitis, but no data has been reported on their ability to mitigate corneal pseudomicrocysts [20•].

Zhao et al. [41] investigated macropinocytosis as a potential culprit of ADC-induced ocular toxicity. Specifically, they studied cell proliferation in human corneal epithelial cell (HCEC) culture exposed to modified versions of ADCs and macropinocytosis inhibitors. ADCs were modified to alter positive charges and hydrophobic residues by attaching polyethylene glycol, polyglutamate residues, or by mutating amino acids on the ADC. In a separate experiment, cells were exposed to unmodified ADCs with and without macropinocytosis inhibitors. In both conditions, cell viability of HCECs increased when treated with macropinocytosis inhibitors or modified ADCs.

Calm Water Therapeutics (Rochester, NY) demonstrated in a pilot study the ability of polylysine-graft-polyethylene glycol (PLL-g-PEG) to inhibit the uptake of ADCs by HCECs in vitro. Specifically, PLL-g-PEG dose dependently decreased ADC uptake in HCECs exposed to rituximab-MMAF, an ADC in development. PLL-g-PEG is proposed to create electrostatic interference between ADCs and off-target cell receptors to mitigate ocular surface toxicities [42]. Future studies are needed to investigate PLL-g-PEG in patients.

Loberg et al. [37•] assessed the corneal toxicity in monkeys caused by depatuxizumab mafodotin (ABT-414). They administered depatuxizumab, the anti-EGFR mAb that is the antibody component of the ADC, systemically and topically via eye drops. Their rationale was to saturate the binding of depatuxizumab to EGFR to inhibit the ADC (Depatuxizumab mafodotin) binding EGFR expressed in the cornea. They also investigated the success of topical preventive therapies, such as vasoconstrictors, tear stimulants/anti-inflammatories, and lubrication with antioxidants, none of which were successful.

Kreps et al. [43] reported the use of 20% autologous serum tear eye drops 6 times daily in one patient being treated with trastuzumab emtansine (Kadcyla) who had developed corneal pseudomicrocysts. Despite ongoing ADC treatment for 14 months, the patient had no worsening of the corneal pseudomicrocysts while using 20% autologous serum tear eye drops.

In our experience, scleral lenses offer an effective solution to improve the eye pain and blurry vision associated with corneal pseudomicrocysts. However, scleral lenses are logistically challenging to utilize given the significant time needed for lens fitting, steep learning curve to place and remove the lenses, and high out-of-pocket expense to patients.

## Conclusion

ADCs are a promising and increasingly utilized targeted cancer therapy that are associated with corneal pseudomicrocysts and various ocular surface AEs. As a result of this increasing popularity of ADCs, there is a need to standardize the reporting and treatment of corneal pseudomicrocysts induced by ADCs. Current preventive therapies for corneal pseudomicrocysts (e.g., topical steroids, vasoconstrictors, PF-ATs) have limited efficacy, but corneal pseudomicrocysts are reversible with modification of ADC therapy, including dose delay, dose reduction, and drug cessation. There is a need for unambiguous ocular surface AE grading scales to ensure accurate and timely detection of ADC-related AEs. This will facilitate clear communication between eye care providers and oncologists to prevent and mitigate ocular surface toxicity through ADC dose modifications. Future research on ADCs is needed to elucidate the mechanisms of ocular surface toxicity and to explore novel therapeutic approaches.

## Figures and Tables

**Table 1:** Ocular Surface Toxicities in FDA-Approved Antibody–Drug-Conjugates (ADCs)

Drug Name	Indication	Antibody (Ocular Surface Expression)	Payload (Mechanism)	Prevalence of corneal pseudomicrocysts	Other Ocular Surface AEs	Ocular Surface Preventive Therapies	Efficacy of Ocular Surface Preventive Therapies	References
Belantamab mafodotin (Blenrep)*	Relapsed or refractory multiple myeloma	BCMA (Not expressed)	MMAF (Microtubule disruption)	68–78%	Keratopathy (76%) Dry eye (19%)	*Prednisolone phosphate 1 %* or *Dexamethasone 0.1 %* eye drops 4x/day for 4–7 days starting 1 day before each infusion Preservative-free artificial tears (PF-ATs) 4x/day from start of infusion	Topical steroids had no effect on preventing corneal changes	[17••, 23, 26, 30, 33, 44–46]
Mirvetuximab soravtansine (Elahere)	Epithelial ovarian, fallopian tube, or primary peritoneal cancer	FRα (Conjunctiva)	DM4 (Microtubule disruption)	41–100%	Keratopathy (29%) Dry eye (25%)	*Prednisolone acetate 1 %* eye drops 4–6x/day for 10 days after infusion *FJuoromethoJone acetate* (FML) eye drops tapered over 4–6 weeks PF-ATs daily	FML for 4–6 weeks cleared corneal pseudomicrocysts and recovered visual acuity *Prednisolone acetate* reduced rate of keratopathy from 41 to 30%	[28,33,38,46,47]
Trastuzumab emtansine (Kadcyla)	HER2 + breast cancer	HER2 (Cornea & conjunctiva)	DM1 (Microtubule disruption)	100%	Dry eye (10%) Conjunctivitis (4%)	Not reported	Not reported	[33, 46, 48, 49]
Tisotumab vedotin (Tivdak)	Recurrent or metastatic cervical cancer	Tissue factor (Cornea & conjunctiva)	MMAE (Microtubule disruption)	Not reported	Conjunctivitis (43%) Conjunctival AEs (40%) Dry eye (23%) Corneal AEs (21%) Keratitis (11%)	*Dexamethasone 0.1 %* eye drops prior to infusion and 3x/day for 3 days after infusion *Brimonidine tartrate 0.2%* eye drops 3 times 10 min prior to infusion PF-ATs as needed until 30 days after last infusion Cold eye masks over each eye during infusion	In an ocular substudy, preventive therapeutic regimen reduced rate of conjunctivitis from 80 to 28% and rate of all ocular AEs from 80 to 60%	[14•, 33, 46, 50–52]
Enfortumab vedotin (Padcev)	Urothelial bladder cancer	Nectin4 (Cornea & conjunctiva)	MMAE (Microtubule disruption)	Not reported	Dry eye (36%)	PF-ATs as needed Topical steroids (dosage and frequency not specified)	Not reported	[8, 33, 46, 53]
Trastuzumab deruxtecan (Enhertu)	HER2 + breast cancer	HER2 (Cornea & conjunctiva)	Camptothecin (DNA damage)	Not reported	Dry eye (11%)	Not reported	Not reported	[33, 46, 54, 55]
Gemtuzumab ozogamicin (Mylotarg)	Newly diagnosed, relapsed, or refractory acute myeloid leukemia	CD33 (Conjunctiva)	Calicheamicin (DNA damage)	NA	NA	NA	NA	[33, 46, 56]
Brentuximab vedotin (Adcetris)	Lymphoma	CD30 (Not expressed)	MMAE (Microtubule disruption)	NA	NA	NA	NA	[33, 46, 57]
Inotuzumab ozogamicin (Besponsa)	Relapsed or refractory B cell acute lymphoblastic leukemia	CD22 (Conjunctiva)	Calicheamicin (DNA damage)	NA	NA	NA	NA	[33, 46, 58]
Polatuzumab vedotin (Polivy)	Relapsed or refractory diffuse large B cell lymphoma	CD79b (Conjunctiva)	MMAE (Microtubule disruption)	NA	NA	NA	NA	[33, 46, 59]
Sacituzumab govitecan (Trodelvy)	Metastatic triplenegative breast cancer; urothelial bladder cancer	TROP2 (Cornea & conjunctiva)	Camptothecin (DNA damage)	NA	NA	NA	NA	[33, 46, 60]
Loncastuximab tesirine (Zynlonta)	Large B cell lymphoma	CD19 (Cornea)	PBD dimer (DNA damage)	NA	NA	NA	NA	(33, 46, 61)

* Withdrawn from US market after failure to meet FDA requirements from DREAMM-3 phase III confirmatory trial
